# Sex-specific prognostic value of triceps skinfold thickness and albumin in pancreatic cancer

**DOI:** 10.1016/j.isci.2026.115290

**Published:** 2026-03-10

**Authors:** Young Hoon Choi, Sang Ah Chi, Kyunga Kim, Jong-In Chang, Hyemin Kim, Dong Kee Jang, Se-Hoon Lee, Jong Kyun Lee, Kyu Taek Lee, Kwang Hyuck Lee, Joo Kyung Park

**Affiliations:** 1 Department of Medicine, Samsung Medical Center, Sungkyunkwan University School of Medicine, Seoul, Republic of Korea; 2 Biomedical Statistics Center, Research Institute for Future Medicine, Samsung Medical Center, Seoul, Republic of Korea; 3 Department of Internal Medicine, Chung-Ang University College of Medicine, Seoul, Republic of Korea; 4 Department of Internal Medicine, Seoul Metropolitan Government Boramae Medical Center, Seoul National University College of Medicine, Seoul, Republic of Korea; 5 Department of Health Sciences and Technology, Samsung Advanced Institute for Health Sciences and Technology (SAHIST), Sungkyunkwan University, Seoul, Republic of Korea; 6 Department of Clinical Research Design and Evaluation, Samsung Advanced Institute for Health Sciences and Technology (SAHIST), Sungkyunkwan University, Seoul, Republic of Korea

**Keywords:** Health sciences, Medicine, Oncology

## Abstract

This study evaluated how triceps skinfold thickness, reflecting body fat, and serum albumin levels, indicating nutritional status, relate to overall survival in patients with pancreatic cancer receiving chemotherapy. We prospectively evaluated 353 patients with pancreatic cancer, repeatedly measuring triceps skinfold thickness and serum albumin from baseline through follow-up. Survival was assessed through a six-month landmark analysis and sex-stratified evaluations. Changes in triceps skinfold thickness, albumin, and protein levels over six months, as well as baseline albumin levels, were independently associated with survival. Cancer stage, chemotherapy response, and six-month CA19-9 levels also contributed to prognosis. Sex-specific analyses showed that reduced triceps skinfold thickness over six months was linked to shorter survival in males, whereas lower baseline albumin levels and greater albumin decline were associated with poorer outcomes in females. These findings highlight the significant and sex-dependent association of triceps skinfold thickness and serum albumin with survival in pancreatic cancer.

## Highlights

• Prospective cohort links TSF and albumin dynamics to survival in pancreatic cancer
• Six-month changes in TSF, albumin, and protein predict survival after chemotherapy
• TSF loss predicts poor survival in males; albumin decline is key in females
• Sex-specific nomograms with TSF and albumin stratify one-to three-year survival

## Graphical abstract

**Figure:**
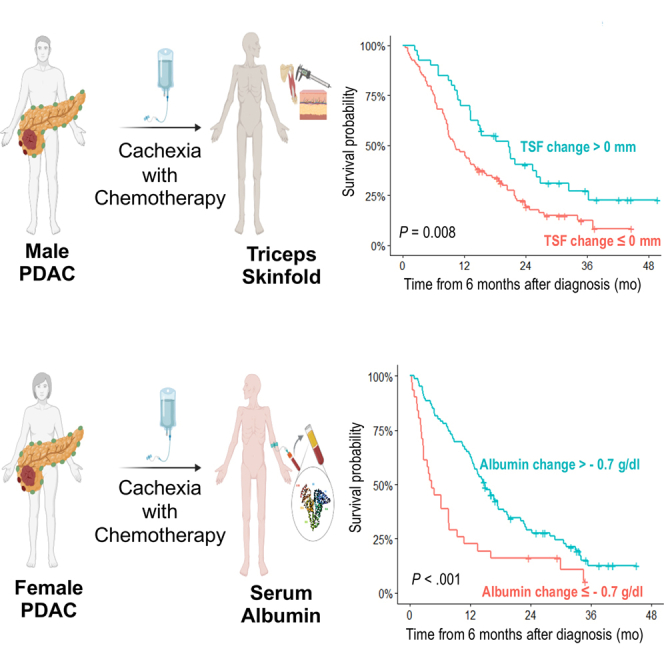

## Introduction

Pancreatic cancer is a lethal malignancy with a five-year survival rate of around 10%.^1^ Several factors, including an advanced stage at diagnosis, elevated serum CA19-9 levels, and poor performance status, have been identified as poor prognostic factors in pancreatic cancer.^2^^,^^3^ Malnutrition and cachexia, prevalent among patients with pancreatic cancer, also contribute to an unfavorable prognosis by impacting treatment tolerance and response.^4^^,^^5^^,^^6^ Serum albumin levels are frequently used as a readily available indicator of nutritional status, and hypoalbuminemia is known as an unfavorable prognostic factor in patients with cancer.^7^^,^^8^ Two studies showed an association between baseline serum albumin levels and overall survival (OS) in patients with pancreatic cancer who received chemotherapy. However, one of these studies focused on patients receiving bevacizumab treatment, which is currently not widely used in pancreatic cancer therapy.^9^ The other study was conducted retrospectively with a small sample size.^10^ Although cachexia and nutritional status are generally expected to deteriorate during the progression of pancreatic cancer, no study has analyzed the relationship between changes in serum albumin levels during pancreatic cancer treatment and survival. Thus, a prospective study to elucidate the connection between initial albumin levels and their alterations during treatment and survival among patients with pancreatic cancer is warranted.

Cachexia is a multifactorial syndrome characterized by weight loss, muscle wasting, and systemic inflammation that occurs in patients with cancer.^5^^,^^6^^,^^11^ In particular, weight loss in patients with pancreatic cancer is mainly attributed to the loss of adipose tissue.^12^ Recent studies reported that extracellular vesicles derived from pancreatic cancer carry integrins specifically targeting adipocytes and induce lipolysis, representing one of the mechanisms contributing to pancreatic cancer-associated cachexia.^13^ Therefore, evaluating fat loss can help assess cachexia in patients with pancreatic cancer, and measuring triceps skinfold (TSF) thickness is one of the simplest methods for assessing body fat. Another study reported that TSF was the most useful among several anthropometric measurements in evaluating the one-year survival of patients with cancer with cachexia.^14^ Considering that the median OS of patients with advanced pancreatic cancer receiving chemotherapy is around one year, TSF is expected to be highly valuable for predicting the prognosis of patients with pancreatic cancer.^15^ However, no studies on the association between changes in TSF during pancreatic cancer treatment and patient survival have been conducted to date. Thus, in the current study, we aimed to examine the potential significant association between baseline and longitudinal changes in serum albumin levels and TSF and OS in patients with pancreatic cancer, using a prospective cohort.

## Results

### Patient characteristics

During the study period, 450 patients were diagnosed with unresectable or borderline resectable pancreatic cancer. After excluding patients with a history or presence of other malignancies (*n* = 5), those who received only the best supportive care (*n* = 57), and those who underwent first-line chemotherapy other than FOLFIRINOX or gemcitabine plus nab-paclitaxel (GnP) (*n* = 35), a total of 353 patients were included in this study (Figure S1). The median age of the 353 patients with pancreatic cancer was 62 years (range, 37–82 years); 151 were females, and 202 were males. The baseline characteristics of the study cohort are summarized in Table 1. Most patients had clinical stage III or IV (*n* = 343, 97.2%). All participants received chemotherapy, of which 23.2% received GnP, and 76.8% underwent a FOLFIRINOX regimen. Females had higher initial and six-month follow-up TSF values than males, whereas albumin and total protein levels were not different between males and females. A total of 292 patients were alive six months after the diagnosis of pancreatic cancer, and their baseline characteristics are shown in Table S1. Of these patients, 240 deaths occurred during a median follow-up of 40.9 months (range, 20.8–55.8 months) (incidence rates were 68.9, 67.7, and 70.4 per 100 person-years in overall, male, and female patients, respectively).

**Table 1:** Baseline characteristics

Variables	Overall (*n* = 353)	Males (*n* = 202)	Females (*n* = 151)	*P*
Body mass index, kg/m^2^				0.124
underweight <18.5	30 (8.5%)	12 (5.9%)	18 (11.9%)	
normal 18.5 to <23	170 (48.2%)	102 (50.5%)	68 (45%)	
overweight ≥23	153 (43.3%)	88 (43.6%)	65 (43%)	
ECOG performance status				0.519
0	187 (53%)	110 (54.5%)	77 (51%)	
1	166 (47%)	92 (45.5%)	74 (49%)	
Smoking				<0.001
Never	199 (56.4%)	58 (28.7%)	141 (93.4%)	
Ex-smoker	94 (26.6%)	86 (42.6%)	8 (5.3%)	
Current smoker	60 (17%)	58 (28.7%)	2 (1.3%)	
Diabetes mellitus				0.156
No	226 (64%)	123 (60.9%)	103 (68.2%)	
Yes	127 (36%)	79 (39.1%)	48 (31.8%)	
Cardiovascular disease				0.205
No	341 (96.6%)	193 (95.5%)	148 (98%)	
Yes	12 (3.4%)	9 (4.5%)	3 (2%)	
Clinical stage, AJCC 8th				0.758
I	8 (2.3%)	4 (2%)	4 (2.6%)	
II	2 (0.6%)	2 (1%)	0 (0%)	
III	154 (43.6%)	87 (43.1%)	67 (44.4%)	
IV	189 (53.5%)	109 (54%)	80 (53%)	
Chemotherapy regimen				0.784
Gemcitabine plus nab-paclitaxel	82 (23.2%)	48 (23.8%)	34 (22.5%)	
FOLFIRINOX	271 (76.8%)	154 (76.2%)	117 (77.5%)	
First line chemotherapy response				0.355
Partial response	128 (36.3%)	67 (33.2%)	61 (40.4%)	
Stable disease	146 (41.4%)	85 (42.1%)	61 (40.4%)	
Progressive disease	46 (13%)	31 (15.3%)	15 (9.9%)	
Not evaluated	33 (9.3%)	19 (9.4%)	14 (9.3%)	
CA 19–9, U/mL				
Initial	270.6 (1.2–486661)	202.2 (1.2–140000)	369.4 (2–486661)	0.021
Six-month^a^	61.7 (2–291132)	42.3 (2–291132)	142.5 (2–140000)	0.001
Total protein, g/dL				
Initial	7 (4.3–8.7)	7.1 (4.3–8.7)	6.9 (5.1–8.6)	0.050
Six-month^a^	7 (4.3–8.6)	7 (4.5–8.5)	6.9 (4.3–8.6)	0.135
Albumin, g/dL				
Initial	4.3 (2.9–5.1)	4.3 (2.9–5.1)	4.3 (2.9–5)	0.484
Six-month^a^	4 (1.4–5.2)	4 (1.4–5.2)	3.9 (2–4.8)	0.136
Triceps skinfold thickness, mm				
Initial	10 (2–42)	8 (2–42)	12 (3–40)	<0.001
Six-month^a^	8 (2–30)	6.4 (2–20)	10 (2.3–30)	<0.001

ECOG, Eastern Cooperative Oncology Group; AJCC, American Joint Committee on Cancer.

a There exist missing values in data: 80 (22.7%), 88 (24.9%), 88 (24.9%), and 116 (32.9%) of the study population for CA 19-9, total protein, albumin, and triceps skinfold thickness measured at six-month, respectively.

### Distribution of TSF and albumin levels

The distribution of initial TSF and initial albumin levels, according to sex and various clinical characteristics, is shown in Figure S2. TSF was significantly higher in females than in males in all subgroups except for the underweight group with a body mass index (BMI) of less than 18.5. Albumin levels were not different according to sex in any subgroups except for the BMI overweight subgroup. No patient characteristics showed significant interactions with sex for initial TSF. In contrast, significant interactions with sex were observed for initial albumin levels on the clinical characteristics of BMI, cancer stage, and CA19-9 levels (Tables S2 and S3).

### Factors associated with overall survival

Univariate analysis showed that Eastern Cooperative Oncology Group (ECOG) performance status, clinical cancer stage, chemotherapy regimen, first-line chemotherapy response, six-month CA19-9 levels, initial albumin, and changes in albumin, protein levels, and TSF over six months were significant indicators of OS. Multivariate analysis revealed independent predictors of OS as follows. An initial albumin less than 3.5 g/dL was associated with shorter OS than an initial albumin greater than 3.5 g/dL (hazard ratio [HR], 2.80; 95% confidence interval [CI], 1.58–4.95, *p* < 0.001). In contrast, initial TSF and initial protein levels were not associated with OS. TSF changes over six months less than the cutoff value of 0 were associated with worse OS compared to TSF changes over six months greater than 0 (HR, 1.51; 95% CI, 1.08–2.12, *p* = 0.017). Similar associations were found for changes in albumin levels over six months (HR, 1.70; 95% CI, 1.13–2.55, *p* = 0.010) and changes in protein levels over six months (HR, 2.03; 95% CI, 1.27–3.23, *p* = 0.003). Clinical cancer stage IV (HR, 1.55; 95% CI, 1.11–2.17, *p* = 0.011), progressive disease after first-line chemotherapy (HR, 3.80; 95% CI, 2.21–6.55, *p* < 0.001), and six-month serum CA19-9 levels greater than 34 U/mL (HR, 2.09; 95% CI, 1.50–2.92, *p* < 0.001) were also independently associated with decreased OS (Table S4).

### Sex differences in the prognostic effects of TSF and albumin

The results of univariate and multivariate survival analyses stratified by sex are shown for males (Table 2) and females (Table 3), respectively. In males, the univariate analysis showed that ECOG performance status, smoking, clinical cancer stage, chemotherapy regimen, first-line chemotherapy response, six-month serum CA19-9 levels, and TSF changes over six months were significant factors for OS. In females, clinical cancer stage, chemotherapy regimen, first-line chemotherapy response, six-month serum CA19-9 levels, initial albumin, and changes in albumin and protein levels over six months were relevant factors for OS in the univariate analysis.

**Table 2:** Factors predicting overall survival in males

	Univariate			Multivariate		
Variables	HR	95% CI	*P*	HR	95% CI	*P*
Age, years (>60 vs. ≤ 60)	1.08	0.84–1.40	0.715	–	–	–
Body mass index, kg/m^2^	–	–	0.836	–	–	–
(Underweight vs. Normal)	0.94	0.56–1.59	0.905	–	–	–
(Overweight vs. Normal)	0.93	0.72–1.21	0.549	–	–	–
ECOG performance status (1 vs. 0)	1.37	1.06–1.77	0.022	1.28	0.83–1.99	0.261
Smoking	–	–	0.096	–	–	0.248
(Ex-smoker vs. Never)	1.24	0.93–1.67	0.032	1.07	0.65–1.74	0.801
(Current smoker vs. Never)	1.07	0.75–1.53	0.149	1.51	0.89–2.54	0.123
Diabetes mellitus (Yes vs. No)	1.02	0.78–1.32	0.419	–	–	–
Cardiovascular disease (Yes vs. No)	0.31	0.08–1.24	0.155	–	–	–
Clinical stage, AJCC 8th (IV vs. I-III)	1.91	1.48–2.46	<0.001	1.69	1.07–2.67	0.025
Chemotherapy regimen						
(FOLFIRINOX vs. Gemcitabine + nab-paclitaxel)	0.61	0.45–0.83	0.042	0.71	0.40–1.28	0.255
First line chemotherapy response	–	–	<0.001	–	–	<0.001
(Stable disease vs. Partial response)	1.36	1.03–1.79	0.037	1.06	0.67–1.69	0.792
(Progressive disease vs. Partial response)	4.32	2.81–6.62	<0.001	3.96	1.98–7.95	<0.001
Initial CA 19–9, U/mL (>34 vs. ≤ 34)	1.26	0.92–1.71	0.302	–	–	–
Six-month CA 19–9, U/mL (>34 vs. ≤ 34)	2.21	1.66–2.95	<0.001	1.92	1.24–2.98	0.004
Initial albumin, g/dL (≤3.5 vs. > 3.5)	1.64	1.01–2.65	0.544	–	–	–
Albumin changes over 6 months, g/dL						
(≤−0.4 vs. > −0.4)	1.76	1.31–2.36	0.077	1.10	0.69–1.76	0.697
Initial protein, g/dL (≤6.4 vs. > 6.4)	1.04	0.75–1.46	0.858	–	–	–
Protein changes over 6 months, g/dL						
(≤0.2 vs. > 0.2)	2.43	1.71–3.47	0.074	1.36	0.85–2.18	0.198
Initial TSF, mm (≤7 vs. > 7)	1.12	0.85–1.49	0.740	–	–	–
TSF changes over 6 months, mm (≤0 vs. > 0)	1.56	1.13–2.16	0.009	1.84	1.14–2.95	0.012

HR, hazard ratio; CI, confidence interval; ECOG, Eastern Cooperative Oncology Group; AJCC, American Joint Committee on Cancer; mo, months; TSF, triceps skinfold thickness.

**Table 3:** Factors predicting overall survival in females

	Univariate			Multivariate		
Variables	HR	95% CI	*P*	HR	95% CI	*P*
Age, years (>60 vs. ≤ 60)	1.07	0.73–1.57	0.728	–	–	–
Body mass index, kg/m^2^	–	–	0.320	–	–	–
(Underweight vs. Normal)	0.83	0.43–1.59	0.566	–	–	–
(Overweight vs. Normal)	0.73	0.49–1.10	0.133	–	–	–
ECOG performance status (1 vs. 0)	1.24	0.84–1.82	0.278	–	–	–
Smoking	–	–	0.678	–	–	–
(Ex-smoker vs. Never)	1.09	0.48–2.49	0.838	–	–	–
(Current smoker vs. Never)	0.42	0.06–3.05	0.394	–	–	–
Diabetes mellitus (Yes vs. No)	1.31	0.86–1.98	0.206	–	–	–
Cardiovascular disease (Yes vs. No)	0.41	0.06–2.97	0.378	–	–	–
Clinical stage, AJCC 8th (IV vs. I-III)	1.82	1.24–2.67	0.002	1.51	0.95–2.41	0.081
Chemotherapy regimen						
(FOLFIRINOX vs. Gemcitabine + nab-paclitaxel)	0.56	0.36–0.88	0.012	0.60	0.34–1.07	0.085
First line chemotherapy response	–	–	<0.001	–	–	0.002
(Stable disease vs. Partial response)	1.22	0.81–1.84	0.332	1.61	0.99–2.63	0.057
(Progressive disease vs. Partial response)	8.51	4.10–17.68	<0.001	4.72	1.93–11.53	0.001
Initial CA 19–9, U/mL (>34 vs. ≤ 34)	1.25	0.76–2.06	0.374	–	–	–
Six-month CA 19–9, U/mL (>34 vs. ≤ 34)	2.38	1.49–3.81	<0.001	2.44	1.47–24.06	0.001
Initial albumin, g/dL (≤3.5 vs. > 3.5)	3.11	1.50–6.46	0.002	5.73	2.48–13.21	<0.001
Albumin changes over 6 months, g/dL						
(≤−0.7 vs. > −0.7)	2.13	1.37–3.33	0.001	3.03	1.51–6.07	0.002
Initial protein, g/dL (≤6.4 vs. > 6.4)	0.87	0.52–1.45	0.589	–	–	–
Protein changes over 6 months, g/dL						
(≤−0.9 vs. > −0.9)	2.43	1.48–3.98	<0.001	1.35	0.65–2.79	0.419
Initial TSF, mm (≤16 vs. > 16)	1.12	0.73–1.70	0.607	–	–	–
TSF changes over 6 months, mm	1.31	0.80–2.14	0.279	–	–	–

HR, hazard ratio; CI, confidence interval; ECOG, Eastern Cooperative Oncology Group; AJCC, American Joint Committee on Cancer; mo, months; TSF, triceps skinfold thickness.

**Figure 1:**
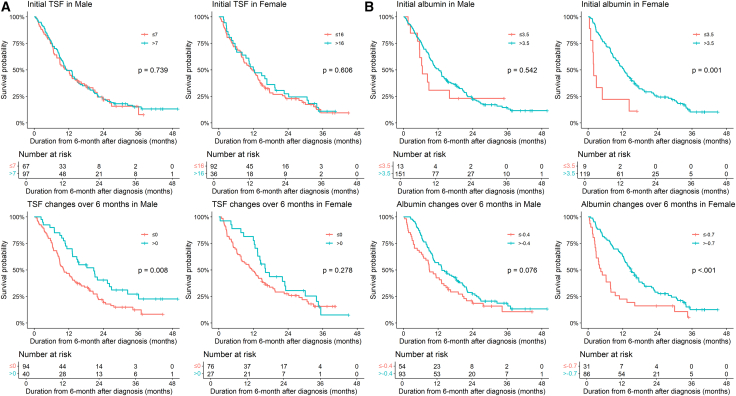
Kaplan-Meier curves for baseline and 6-month changes in TSF and albumin Kaplan-Meier curves based on the initial values and changes over six months in (A) TSF (mm) and (B) albumin levels (g/dL). Survival curves were compared using the two-sided log rank test. (A) Initial TSF in males: ≤7 (*n* = 67) versus >7 (*n* = 97), *p* = 0.739. Initial TSF in females: ≤16 (*n* = 92) versus >16 (*n* = 36), *p* = 0.606. TSF changes over 6 months in males: ≤0 (*n* = 94) versus >0 (*n* = 40), *p* = 0.008. TSF changes over 6 months in females: ≤0 (*n* = 76) versus >0 (*n* = 27), *p* = 0.278. (B) Initial albumin in males: ≤3.5 (*n* = 13) versus >3.5 (*n* = 151), *p* = 0.542. Initial albumin in females: ≤3.5 (*n* = 9) versus >3.5 (*n* = 119), *p* = 0.001. Albumin changes over 6 months in males: ≤ −0.4 (*n* = 54) versus > −0.4 (*n* = 93), *p* = 0.076. Albumin changes over 6 months in females: ≤ −0.7 (*n* = 31) versus > −0.7 (*n* = 86), *p* < 0.001. TSF, triceps skinfold thickness.

The results of the multivariate survival analysis were as follows. For males, TSF changes over six months less than the cutoff value of 0 increased the risk of death compared to TSF changes over six months greater than the cutoff value (HR, 1.84; 95% CI, 1.14–2.95, *p* = 0.012). However, initial albumin levels and changes in albumin levels over six months were not associated with OS in males. In contrast to males, in females, initial albumin less than 3.5 g/dL (HR, 5.73; 95% CI, 2.48–13.21, *p* < 0.001) and changes in albumin levels over six months less than the cutoff value of −0.7 (HR, 3.03; 95% CI, 1.51–6.07, *p* = 0.002) were independently associated with decreased OS. In contrast, TSF changes over six months were not associated with OS (Figure 1). In both males and females, progressive disease after first-line chemotherapy and six-month CA19-9 levels greater than 34 U/mL were associated with poor OS. Clinical cancer stage IV was significantly associated with decreased OS in males, and although not statistically significant, it showed a tendency toward shorter OS in females.

A sensitivity analysis using multiple imputation yielded generally consistent results (Tables S5 and S6). In females, Initial albumin and six-month changes in albumin remained significantly associated with OS, whereas in males, the association between six-month TSF changes and OS showed a similar effect size but did not reach statistical significance.

### Sex-specific nomograms predicting overall survival

We generated sex-specific nomograms of the significant variables in multivariate analysis for OS in subgroups of males and females to address sex-specific composite prognostic predictions (Figure S3). For the females’ nomogram, we also included clinical cancer stage as a variable, which tended to be associated with survival, although not statistically significant in the multivariate analysis for OS in the female subgroup. The C-indices of the nomograms for males and females were 0.69 (95% CI, 0.63–0.74) and 0.74 (95% CI, 0.69–0.79), respectively. In our internal validations, the C-indices for these nomograms were 0.70 (95% CI, 0.69–0.70) and 0.75 (95% CI, 0.74–0.75), respectively. The patients were stratified into three risk groups based on the sum of nomogram points: low (≤5 points), medium (>5 points and ≤10 points), and high (>10 points). Among males, the low, medium, and high-risk groups had median OS times of 33.6 months (95% CI, 26.9–55.8), 16.8 months (95% CI, 14.8–23.5), and 14.3 months (95% CI, 12.5–16.4), respectively (*p* < 0.001). The HR for death was 2.88 (95% CI, 1.72–4.84), 1.90 (95% CI, 1.22–2.96), and 5.48 (95% CI, 3.16–9.53) in the medium versus low, high versus medium, and high versus low-risk groups, respectively (*p* < 0.001).

**Figure 2:**
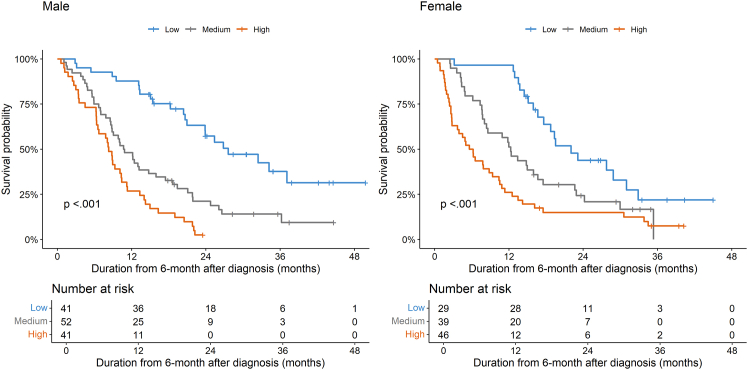
Kaplan-Meier curves for risk groups stratified according to nomogram points in males and females. Survival curves were compared using the two-sided log rank tests. For males, low-risk (*n* = 41), medium-risk (*n* = 52), and high-risk (*n* = 41), *p* < 0.001. For females, low-risk (*n* = 29), medium-risk (*n* = 39), and high-risk (*n* = 46), *p* < 0.001

In females, the low, medium, and high-risk groups had median OS times of 28.0 months (95% CI, 23.6–38.9), 18.3 months (95% CI, 14.4–23.5), and 11.9 months (95% CI, 9.7–16.4), respectively (*p* < 0.001). The HR for death was 1.90 (95% CI, 1.08–3.37), 1.68 (95% CI, 1.06–2.67), and 3.19 (95% CI, 1.84–5.53) in the medium versus low, high versus medium, and high versus low-risk groups, respectively (*p* < 0.001) (Figure 2).

Figure S4 shows the calibration performance of the risk groups in males and females. For both males and females, the integrated calibration index (ICI) and the E50 values were less than 0.1 for one, two, and three-year OS. This indicates that the sex-specific risk groups were well-calibrated for one-, two-, and three-year OS.

## Discussion

This was the first study to evaluate the association between changes in TSF and serum albumin levels over six months and survival using a prospective cohort of patients with pancreatic cancer. Our study revealed that six-month changes in TSF, serum albumin levels, and initial albumin levels were independently associated with OS. Sex differences were evident in these prognostic factors, as TSF emerged as an effective predictor for males, while albumin levels proved effective in predicting outcomes for females. We also developed sex-specific nomograms, incorporating other significant prognostic factors derived from multivariate analysis, to conduct a comprehensive prognostic evaluation. These nomograms allowed us to classify patients into three distinct risk groups based on the nomogram points for each sex, revealing significant differences in OS among the groups. Furthermore, the estimation of one-, two-, and three-year survival rates using the variables derived from sex-specific nomograms demonstrated good performance.

Cachexia occurs frequently in patients with pancreatic cancer, affecting approximately 80% of the patients.^16^ Cancer cachexia has been characterized as involuntary weight loss, particularly skeletal muscle mass loss with or without fat mass loss.^11^ Accordingly, many studies have been conducted on sarcopenia and pancreatic cancer survival.^17^ However, few studies related to adipose tissue loss and pancreatic cancer survival have been conducted. Since lipolysis is essential in the progression of cancer cachexia and even precedes skeletal muscle mass loss, adipose tissue loss in cancer cachexia deserves attention.^18^ Adipose tissue is classified into two types: white adipose tissue (WAT), which stores energy, and brown adipose tissue, which contributes to thermogenesis.^19^ During cancer cachexia, especially in interleukin-6-mediated chronic inflammation, WAT lipolysis and WAT browning occur, contributing to increased energy expenditure and WAT loss.^20^^,^^21^^,^^22^ Since WAT is abundantly distributed in subcutaneous fat, when cancer cachexia causes WAT loss, TSF, an anthropometric measure reflecting subcutaneous fat, may decrease.^23^ In this context, the results of this study suggest that a decrease in six-month TSF from the initial value was associated with poor survival, suggesting that patients with decreased TSF are in the process of cachexia, resulting in a shorter OS. To our knowledge, this study represents initial evidence illustrating the association between changes in TSF and the survival outcomes of patients with pancreatic cancer. Although no previous studies of the relationship between TSF and survival in patients with pancreatic cancer have been conducted, two small studies reported that adipose tissue loss, measured using computed tomography images, was associated with poor prognosis.^24^^,^^25^ However, in these studies, only visceral adipose tissue (VAT) loss, not subcutaneous adipose tissue (SAT) loss, was associated with survival, which was different from the present study. According to the previous report, VAT consistently decreases with cancer progression, while SAT loss gradually accelerates.^26^ This suggests that SAT loss might be prominent in patients with more advanced pancreatic cancer. Since this study included patients with more advanced pancreatic cancer than the two studies mentioned above related to VAT,^24^^,^^25^ changes in TSF, which are related to SAT, in this study may have been significantly associated with survival. In the analysis by sex, the six-month TSF change was significantly associated with survival only in males. A previous study on patients with cachexia from various cancers also showed results consistent with our study, that TSF was more prognostic in males than in females.^27^ Several biological mechanisms may underlie this sex-specific pattern. First, adipose tissue distribution and its response to catabolic stimuli differ between males and females. Males typically have a lower proportion of subcutaneous fat and more VAT, whereas females accumulate more subcutaneous fat under the influence of estrogens.^28^ Consequently, a given absolute reduction in subcutaneous fat, captured by a decline in TSF, may represent a more advanced stage of fat depletion and cancer cachexia in males than in females, making TSF loss a more sensitive marker of adverse prognosis in male patients. Second, accumulating preclinical and clinical evidence indicates sexual dimorphism in cancer-associated cachexia, with males exhibiting greater weight loss, muscle wasting, and a more pronounced systemic inflammatory response.^29^ Pro-inflammatory cytokines such as interleukin-6 (IL-6) and tumor necrosis factor-α (TNF-α) are central mediators of cancer cachexia, promoting WAT lipolysis and driving a highly catabolic metabolic state.^29^ In this context, rapid TSF loss in males may serve as a surrogate marker of an IL-6– and TNF-α-driven inflammatory cachexia phenotype. In contrast, survival in females may be more closely linked to the downstream systemic consequences of inflammation rather than isolated measures of subcutaneous fat loss. Through inflammatory and acute-phase signaling, IL-6 and TNF-α suppress hepatic albumin synthesis.^30^ As a result, serum albumin serves as a composite marker of inflammatory burden, nutritional imbalance, and hepatic metabolic adaptation; notably, several studies have reported lower and more labile albumin levels in women.^31^^,^^32^^,^^33^

Serum albumin synthesis is suppressed under conditions, such as malnutrition and inflammation, that can occur during cancer progression. Accordingly, studies suggested that pretreatment serum albumin levels reflect the prognosis of patients with various cancers.^34^^,^^35^ Most studies on the prognostic value of serum albumin levels in pancreatic cancer have been conducted by including serum albumin levels as a component of the prognostic score rather than evaluating it alone.^36^^,^^37^^,^^38^ One previous study investigated the prognostic value of serum albumin levels alone in advanced pancreatic cancer and reported that baseline serum albumin levels were associated with OS in patients treated with GnP,^10^ consistent with our findings. In addition to baseline serum albumin levels, we showed a significant relationship between changes in serum albumin levels at six months and OS. Our study demonstrated a meaningful relationship between changes in serum albumin levels and OS in patients with pancreatic cancer. Furthermore, a sex-based analysis revealed that the relationship between serum albumin levels and OS was significant solely among females. The prognostic association of changes in albumin levels by sex in patients with pancreatic cancer or other cancers has not been previously reported. In studies related to changes in albumin levels and sex, not limited to cancer, one study reported that changes in albumin levels in patients who underwent major colorectal surgery were more significant in females and were associated with complications.^31^^,^^32^ Although the mechanism is not clear, it can be assumed that changes in serum albumin levels according to the patient’s condition are more likely to occur in females. The results of the interaction analysis in this study, showing sex differences in the extent to which BMI, cancer stage, and serum CA19-9 levels affected serum albumin levels, also partially support this assumption.

We also confirmed that previously known prognostic predictors, including cancer stage, response to chemotherapy, and serum CA19-9 levels during chemotherapy, were associated with OS in this study.^39^^,^^40^^,^^41^ These three factors can be considered to be related to tumor burden. In contrast, the other factors associated with survival in this study, TSF or albumin-related factors, can be regarded as factors reflecting patient conditions related to cancer cachexia, chronic inflammation, and malnutrition. Since males and females are inherently different in physiology, hormones, anatomy, immunity, and so forth,^42^^,^^43^ we believe that the factors representing the condition of patients, such as changes in TSF or serum albumin levels, also differed according to sex in this study. Reflecting on this, we developed a sex-specific nomogram and were the first to suggest the need to tailor the prediction of pancreatic cancer prognosis according to sex. However, this needs to be verified through future studies. In particular, the nomograms were internally validated only through bootstrapping, and external validation in independent cohorts, such as multi-center prospective registries, is necessary to establish their robustness and clinical applicability. Moreover, although TSF is a simple anthropometric measure, obtaining reliable and standardized measurements in routine clinical practice can be challenging. In our study, TSF assessments were performed by two well-trained nurses and repeated three times to minimize inter-observer variability, underscoring the importance of standardized training and protocols for applying TSF-based assessments in real-world clinical settings. Given the potential challenges in applying standardized TSF measurement protocols across diverse clinical settings, future studies are needed to explore whether bioimpedance-based body fat assessment may be used as an alternative surrogate for TSF.

We further examined the potential impact of missing TSF, albumin, and protein levels at 6 months using multiple imputation as a sensitivity analysis. The overall patterns of the results were consistent across the primary and sensitivity analyses, with albumin-related factors in females remaining significant predictors of survival. Although the association between six-month TSF changes and OS in males showed a comparable effect size, it did not reach statistical significance after imputation, suggesting that the precision of sex-specific estimates—particularly in males—may be sensitive to missing-data assumptions. Nonetheless, the primary conclusions regarding the prognostic relevance of longitudinal nutritional changes were broadly robust across analytic approaches.

### Limitations of the study

This study had some limitations. First, the study cohort consisted predominantly of East Asian individuals, which may limit the generalizability of our findings to other racial groups. External validation in multi-center, multi-ethnic cohorts will therefore be required. In addition, because of the observational design, the findings reflect associations rather than causality, and future interventional studies are needed to clarify these relationships. Second, this study did not evaluate factors related to traditional cachexia evaluation, such as body weight change and measures such as mid-arm circumference used for evaluating skeletal muscle mass.^11^ However, recent reports have shown that adipose tissue loss, rather than skeletal muscle loss, is more closely associated with cachexia and poor prognosis in pancreatic cancer.^12^^,^^25^ In line with this, a previous study of patients receiving FOLFIRINOX chemotherapy demonstrated cachexia phenotypes characterized by fat loss with or without concomitant muscle loss.^44^ Therefore, focusing on fat loss alone, as in our study, may be sufficient to identify the majority of patients with pancreatic cancer with cachexia features. Finally, patients who died or were censored within six months of pancreatic cancer diagnosis were excluded from the survival analysis, which may introduce survivorship bias and narrow the range of patients to whom our prognostic factors can be applied. This exclusion was necessary to evaluate longitudinal changes in TSF and serum albumin. These measurements are inherently unavailable for patients who die early and cannot be reliably imputed without strong assumptions. To address this limitation, we additionally performed a sensitivity analysis including all patients from diagnosis and found that the overall patterns of association remained largely consistent (Tables S7 and S8). However, in this sensitivity analysis, the association between six-month TSF change and survival in males was attenuated. This suggests that the prognostic value of TSF change may depend on clinical context and follow-up duration, and may be more informative among patients who survive beyond six months after diagnosis, when longitudinal nutritional changes can be more reliably assessed.

In conclusion, this study was the first to demonstrate an association between survival and changes in TSF, reflecting adipose tissue alterations, as well as baseline and changes in serum albumin levels, in patients with advanced pancreatic cancer undergoing chemotherapy in a prospective cohort. Furthermore, this study revealed sex differences in the prognostic relevance of TSF in males and serum albumin levels in females. Based on this, sex-specific prognostic nomograms were developed, demonstrating distinct survival stratifications across three risk groups based on nomogram points. These findings highlight the importance of sex-specific prognostic predictions. Further research on the underlying mechanisms contributing to these sex differences and validation studies in a larger sample size is needed to corroborate these findings.

## Resource availability

### Lead contact

Requests for further information and resources should be directed to and will be fulfilled by the lead contact, Joo Kyung Park (mdsophie@gmail.com).

### Materials availability

This study did not generate new unique reagents. Please contact the lead contact for further information regarding resources.

### Data and code availability

• This paper does not report any original code.
• Any additional information required to reanalyze the data reported in this paper is available from the lead contact upon reasonable request.

## Acknowledgments

The authors would like to express their great appreciation to the late DJ. Park, HJ. Kim, and the late YA. Holland. We believe their valuable donation would make a tremendous difference in the treatment and survival of such a devastating disease.

This work was supported by the 10.13039/501100001321National Research Foundation of Korea funded by the 10.13039/501100014188Ministry of Science and ICT (grant no. RS-2024-00440814). This research was supported by the Bio&Medical Technology Development Program of the National Research Foundation (NRF) funded by the Korean government (MSIT) (grant no. RS-2023-00225255). This study was supported by the Future Medicine 20∗30 project of the 10.13039/501100024838Samsung Medical Center (grant no. SMX1250111). This research was supported by the National Institute of Health (NIH) research project (project No. 2025-ER1104-01).

## Author contributions

Y.H.C., S.A.C., and K.K. are co-first authors and contributed equally to this work; K.H.L. and J.K.P. are co-correspondents; conceptualization, J.K.P.; methodology, Y.H.C., S.A.C., J.-I.C., K.K., and J.K.P.; data curation, Y.H.C., S.A.C., J.-I.C., and H.K.; investigation, J.-I.C. and J.K.P.; software, Y.H.C. and S.A.C.; resources, J.K.L., K.T.L., K.H.L., and J.K.P.; formal analysis, Y.H.C., S.A.C., and K.K.; visualization, S.A.C. and H.K.; writing – original draft, Y.H.C., S.A.C., and K.K.; writing – review and editing, Y.H.C., S.A.C., K.K., J.-I.C., H.K., D.K.J., S.-H.L., J.K.L., K.T.L., K.H.L., and J.K.P.; funding acquisition, S.-H.L. and J.K.P.; supervision, K.H.L., K.K., and J.K.P.; project administration, K.H.L. and J.K.P.

## Declaration of interests

The authors declare no competing interests.

## STAR★Methods

### Key resources table

**Table:** 

REAGENT or RESOURCE	SOURCE	IDENTIFIER
**Software and algorithms**		

R software (version 4.2.2)	R Foundation for Statistical Computing	N/A

### Experimental model and study participant details

#### Study design and participants

This was a prospective cohort study of pancreatic cancer patients enrolled in a hospital-based prospective registry at Samsung Medical Center (SMC) in Korea. The corresponding protocol was registered on ClinicalTrials.gov (NCT03637569). The present study included patients newly diagnosed with unresectable or borderline resectable pancreatic cancer between April 2018 and June 2021. The exclusion criteria were as follows: 1) patients with a history or presence of a malignant disease other than pancreatic cancer; 2) patients with malabsorption due to pre-existing intestinal disorders; and 3) patients with eating disorders due to pre-existing psychiatric disorders. We also excluded patients who received only the best supportive care or first-line chemotherapy other than FOLFIRINOX or GnP. Finally, only patients who underwent first-line chemotherapy with either FOLFIRINOX or GnP were analyzed. We restricted the analysis to these regimens to reduce treatment-related heterogeneity, as FOLFIRINOX and GnP are established first-line treatments for advanced pancreatic cancer. The Institutional Review Board at SMC approved the research protocol (No. 2018-04-087). The study was conducted following the principles of the Declaration of Helsinki, and all participants provided written informed consent before enrollment.

### Method details

#### Data collection

Anthropometric measurements, including height, weight, BMI, and TSF, were collected according to recommended standardized protocols^45^^,^^46^ during the initial physical examination. TSF was measured at the posterior midpoint of the right arm, between the acromion and olecranon, with the arm freely extended in a standing position, using a plastic skinfold caliper with an accurate measurement up to a maximum of 70.0 mm (Zhong Yan Co.). To improve data reliability, (1) TSF assessment was carried out by two well-trained nurses; and (2) TSF was measured three times to record the mean of these measurements as the TSF value for each assessment. TSF assessment was conducted at diagnosis and subsequently at three-week intervals. BMI was classified into three categories according to Asia-Pacific BMI classifications: underweight (<18.5 kg/m^2^), normal weight (18.5–22.9 kg/m^2^), and overweight (>23 kg/m^2^).^47^

We collected clinical information including age, sex, date of diagnosis, date of death, and major comorbidities such as diabetes and cardiovascular disease. Smoking history was also recorded. The clinical cancer stage at diagnosis was determined according to the 8th edition of the American Joint Committee on Cancer Staging System,^48^ and ECOG performance status^49^ at diagnosis was documented. The initial chemotherapy regimen and the best response to first-line chemotherapy based on Response Evaluation Criteria in Solid Tumors (RECIST) criteria version 1.1^40^ were also recorded. Laboratory parameters, including serum protein, albumin, and CA19-9 levels were measured at diagnosis and every three weeks thereafter. Six-month changes in TSF, serum protein, and albumin levels were obtained by subtracting the initial values from the six-month values. Given the approximate six-month median progression-free survival in pancreatic cancer patients undergoing chemotherapy with FOLFIRINOX or GnP regimen,^15^^,^^50^ the six-month landmark was selected in advance as an appropriate time to evaluate changes in TSF and serum albumin levels related to cancer progression during chemotherapy.

#### Primary outcome

The primary outcome of interest was OS and its association with TSF and serum albumin levels. OS was measured as the duration from the date of pancreatic cancer diagnosis to either the date of death or December 5, 2022, whichever occurred first. Patients were described as censored if they were alive until December 5, 2022.

#### Data preparation and processing

At the six-month assessment, missingness ranged from 22.7% to 32.9% across CA19-9, total protein, albumin, and TSF measurements (Table 1). We used linear interpolation to impute missing values based on observed values between the previous and subsequent time points to reduce the number of patients with missing data on six-month TSF, protein, and albumin levels. The linear interpolation assumes that changes in these levels between adjacent time points follow an approximately linear trend and that the missingness mechanism is close to missing completely at random. To assess the robustness of these assumptions, we additionally conducted a sensitivity analysis using multiple imputation by chained equation under a missing at random assumption. Covariates for the imputation model were selected in accordance with established recommendations, prioritizing variables that were statistically associated with the incomplete nutritional measurements (six-month TSF, albumin, and protein).^51^^,^^52^ We finally chose clinically meaningful ones among these variables. Accordingly, the imputation model included BMI, first-line chemotherapy response, initial TSF, initial albumin, and initial protein levels. The imputation model included BMI, first-line chemotherapy response, initial TSF, initial albumin, and initial protein levels, which were selected based on their clinical relevance and their associations with overall survival or missingness. Six-month TSF, protein, and albumin values were imputed using predictive mean matching. Five imputed datasets were generated, and the estimates were combined using Rubin’s rules. If no observed values were available after six months, missing values were left unimputed. Logarithmic and exponential transformation were used for initial TSF and initial albumin levels, respectively, which had skewed distributions. Some continuous variables were converted to binary variables by optimal cutoff points for initial TSF values and six-month changes in TSF, albumin, and protein levels. The optimal cutoff points with maximum concordance index (C-index) values were selected. The optimal cutoff value for initial TSF was 7 mm, 7 mm, and 16 mm for the entire patient cohort, males, and females, respectively. In the overall patient group, the optimal cutoffs for the six-month changes in TSF, albumin, and protein levels were 0, -0.6, and −0.9, respectively. For the same variables, the optimal cutoffs by sex were 0, -0.4, and 0.2 for males and 0, -0.7, and −0.9 for females.

### Quantification and statistical analysis

Numeric patient characteristics are presented as medians with interquartile ranges or means with standard deviations and were compared between sex groups using the Wilcoxon rank-sum test or the two-sample t-test, as appropriate, based on normality assumption. Categorical variables are described as frequencies with percentages and were compared between groups using the chi-squared test or Fisher’s exact test if the expected cell frequency was <5.

In subgroups based on each clinical characteristic, sex-specific distributions of initial TSF and initial albumin were visualized using side-by-side box plots and compared with the Wilcoxon rank-sum test. The interaction effects between sex and each of the clinical factors on the initial TSF and initial albumin levels were examined using a linear regression model.

We estimated conditional OS using a landmark analysis among patients who were alive six months after the date of pancreatic cancer diagnosis to assess the influence of longitudinal changes in TSF and serum albumin levels on survival. Conditional OS probabilities were estimated using the Kaplan-Meier method and was compared between groups using the log-rank test. Conditional Cox proportional hazard models were used to estimate HRs with 95% CIs. Variables with a *P*-value of less than 0.1 in the univariable analysis were included in the multivariable analysis. Multicollinearity was checked using the variance inflation factor (VIF). There are no variables with VIFs over 5. The proportional hazards assumption was visually inspected by Schoenfeld residuals. No severe violation was observed.

We constructed a nomogram for patients who were alive six months after the date of diagnosis to predict one-, two-, and three-year OS probabilities by including variables with a *P*-value of less than 0.05 in the multivariable analysis. One, two, and three-year OS probabilities were defined as the probability of a patient surviving an additional one, two, and three years, respectively. The nomogram was internally validated using a resampling method with 100 bootstrap samples of the same size as the original data. The discrimination performance of the nomogram was assessed using C-index. The patients were also divided into three risk groups based on individual total points calculated from the nomogram: low-, medium-, and high-risk groups. We used the ICI and the E50 values to assess the calibration of the risk groups, which described the agreement between estimated and observed one-, two-, and three-year survival probabilities. Estimated survival probabilities were calculated from the multivariable Cox regression model with the variables used to construct the nomogram. Statistical significance was set at a 2-sided *P*-value <0.05. *P*-values and sample sizes for each comparison are reported in the corresponding figure legends. All statistical analyses were performed using R statistical software (version 4.2.2; Foundation for Statistical Computing).

#### Additional resources

ClinicalTrials.gov: NCT03637569 (https://clinicaltrials.gov/study/NCT03637569).
