# Supplementary material for: Sex-specific prognostic value of triceps skinfold thickness and albumin in pancreatic cancer
Source: iScience. 2026 Mar 10;29(4):115290. doi: 10.1016/j.isci.2026.115290 (PMC13019937; doi:10.1016/j.isci.2026.115290)
Supplement: Document S1. Figures S1–S4 and Tables S1–S8 [file mmc1.pdf]

iScience, Volume *29*

Supplemental information

Sex-specific prognostic value of triceps skinfold thickness and albumin in pancreatic cancer

Young Hoon Choi, Sang Ah Chi, Kyunga Kim, Jong-In Chang, Hyemin Kim, Dong Kee Jang, Se-Hoon Lee, Jong Kyun Lee, Kyu Taek Lee, Kwang Hyuck Lee, and Joo Kyung Park

Figure S1. Study flow chart.

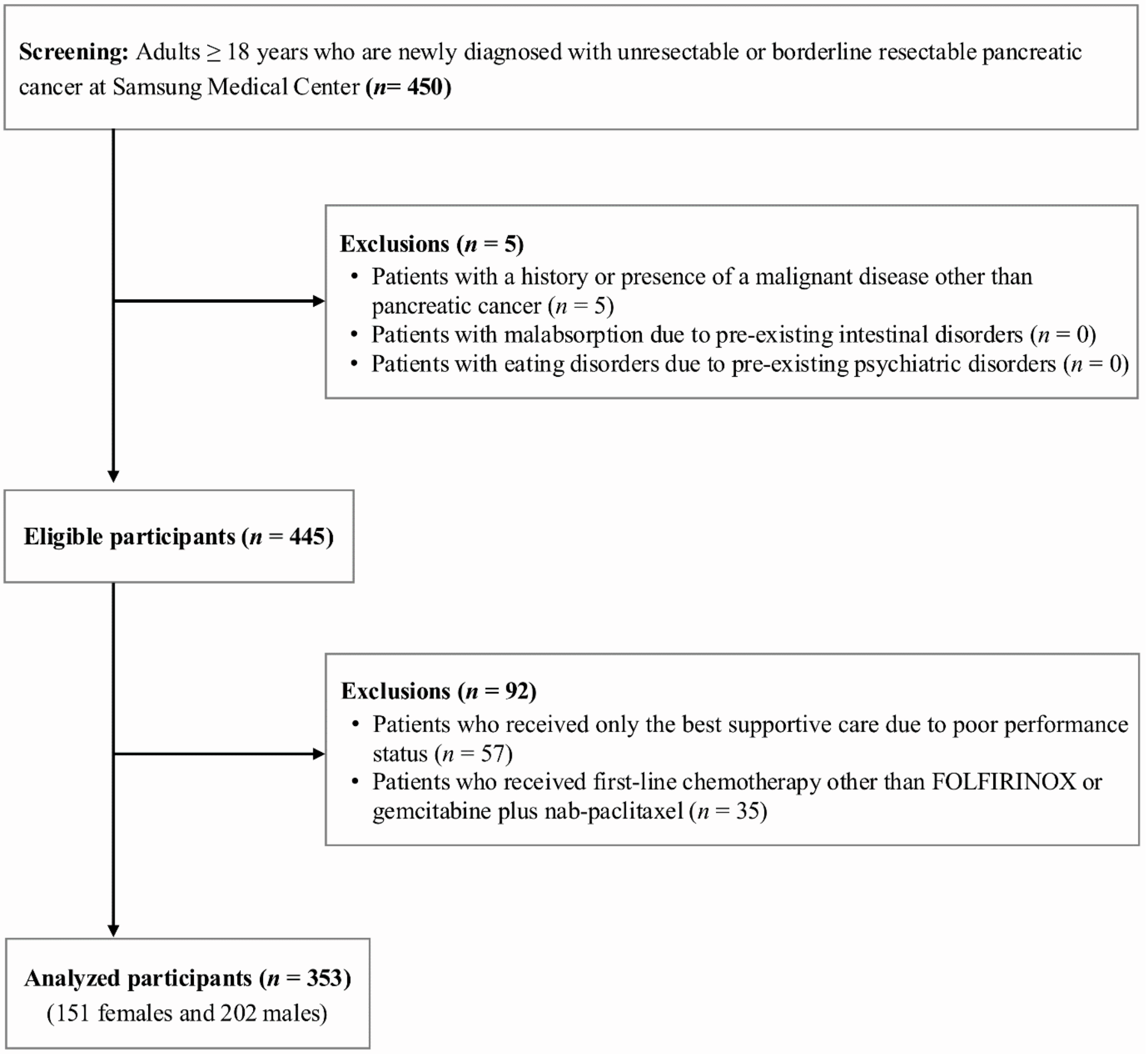

Figure S2. Distribution of (A) initial TSF and (B) initial albumin levels according to sex and clinical characteristics. Data are presented as median (interquartile range). Boxes represent the interquartile range (IQR), center lines indicate medians, and whiskers extend to 1.5 × IQR. Points beyond the whiskers represent outliers. ECOG, Eastern Cooperative Oncology Group; Gem, gemcitabine; TSF, triceps skinfold thickness. See also Table S2 and Table S3.

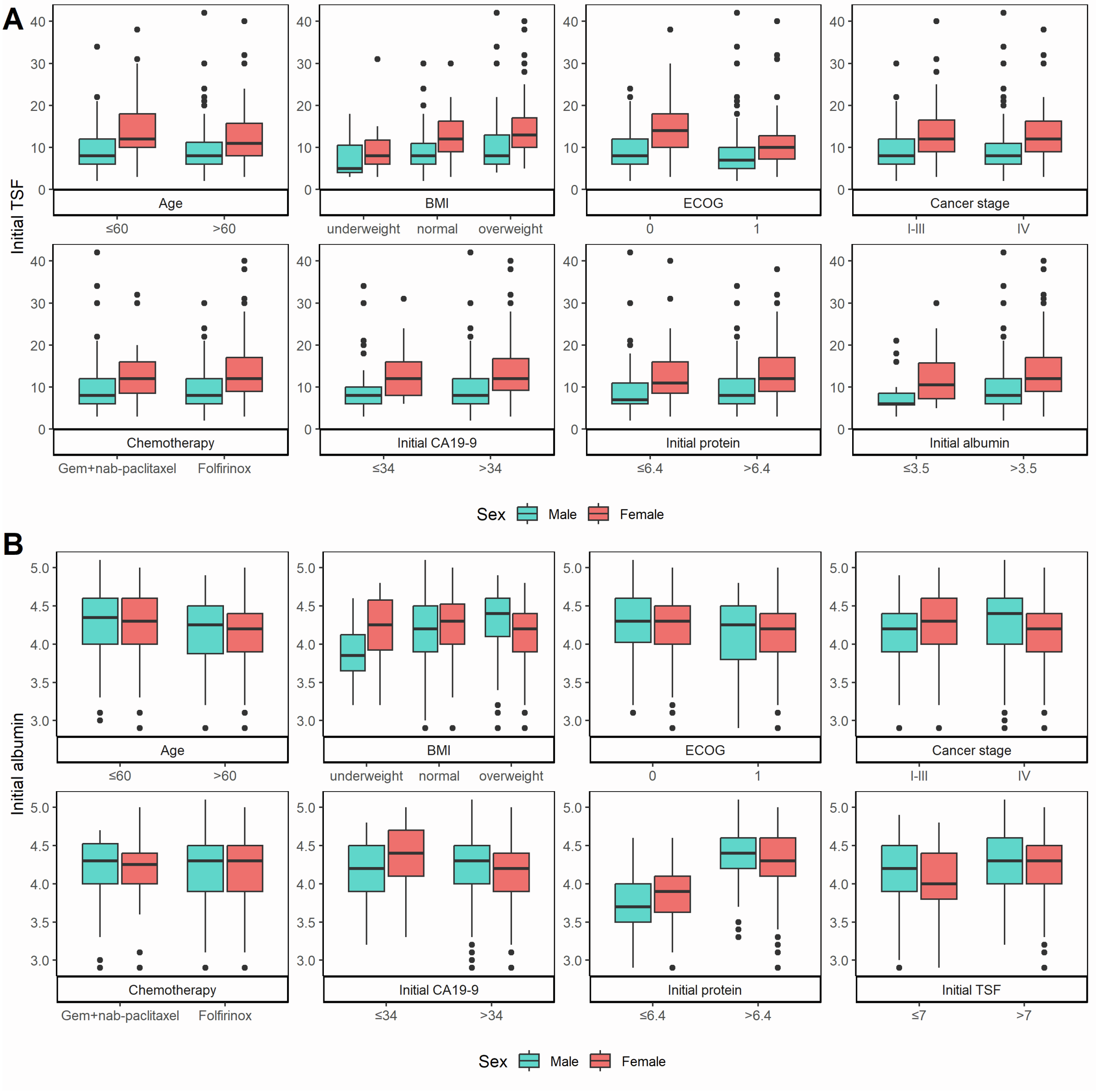

Figure S3. Sex-specific nomograms predicting overall survival in (A) males, and (B) females.

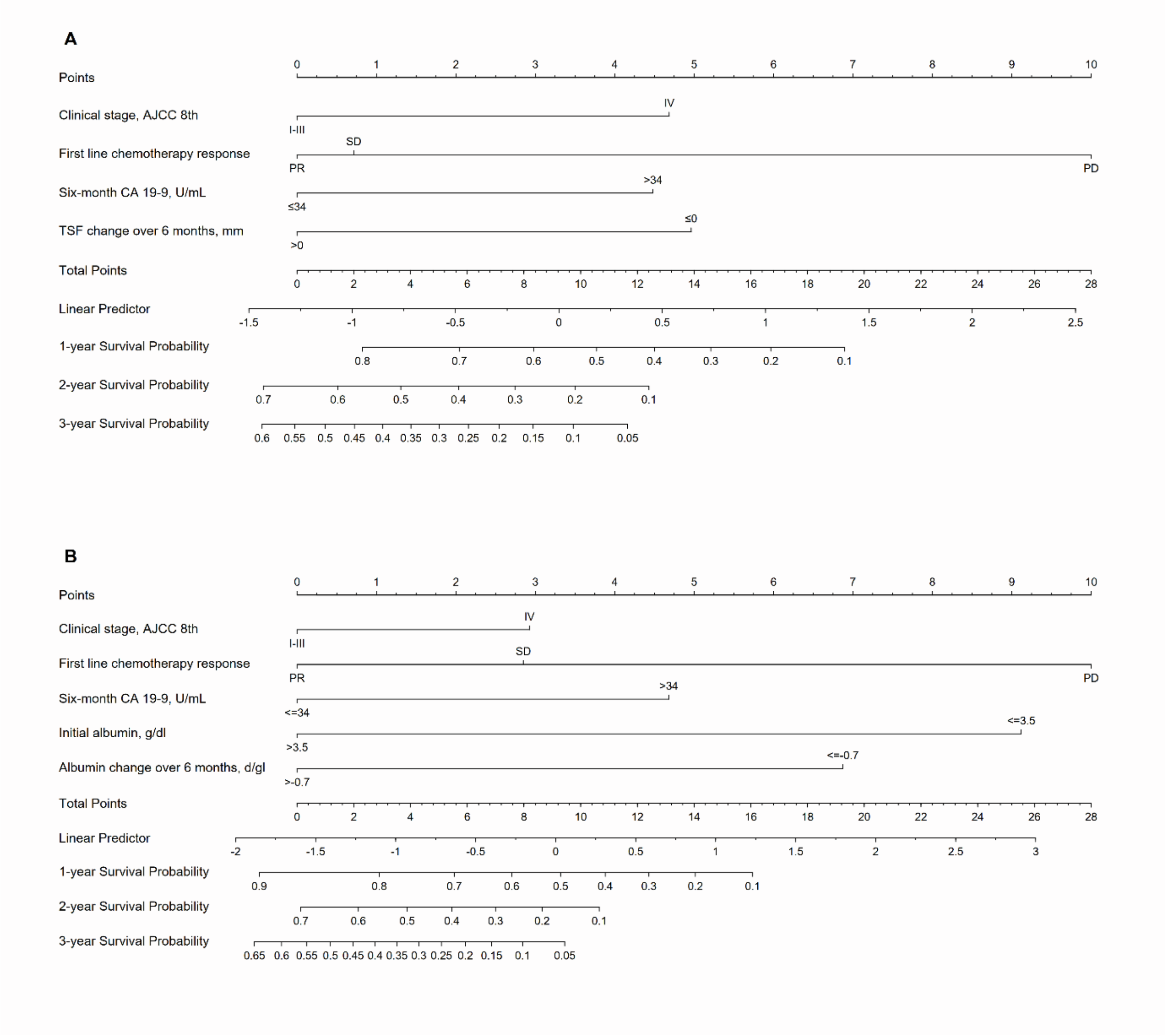

Figure S4. Estimated and observed one-, two-, and three-year survival probabilities for the three risk groups stratified by sex-specific nomogram points in (A) males and (B) females.

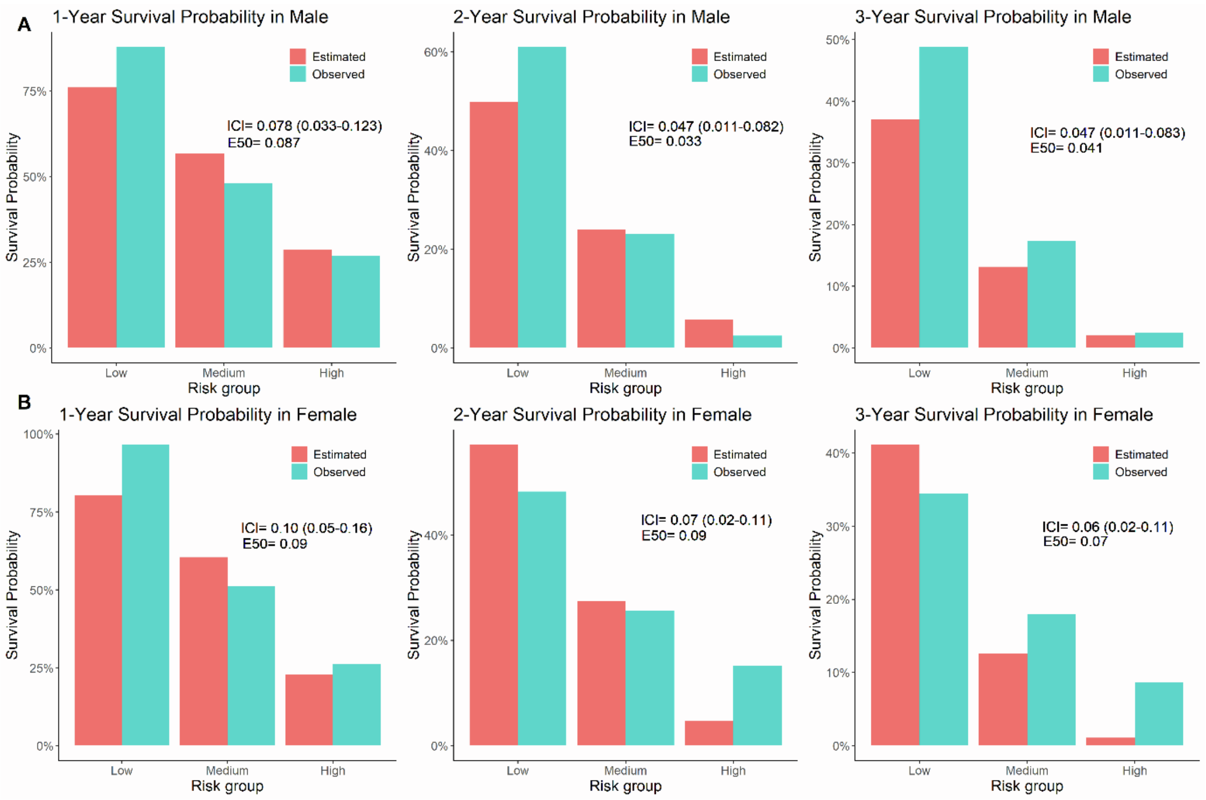

Table S1. Baseline characteristics of patients surviving for over 6 months

Variables	Those alive at 6mo (*n* = 292)	Males alive at 6mo (*n* = 164)	Females alive at 6mo (*n* = 128)	*P*
Age, years	61 (37-82)	61 (37-79)	61 (38-82)	0.442
Body mass index, kg/m^2				0.042
underweight < 18.5	20 (6.8%)	6 (3.7%)	14 (10.9%)	
normal 18.5 to < 23	137 (46.9%)	82 (50%)	55 (43%)	
overweight ≥ 23	135 (46.2%)	76 (46.3%)	59 (46.1%)	
ECOG performance status				0.323
0	168 (57.5%)	99 (60.4%)	69 (53.9%)	
1	124 (42.5%)	65 (39.6%)	59 (46.1%)	
Smoking				<.001
Never	164 (56.2%)	46 (28%)	118 (92.2%)	
Ex-smoker	81 (27.7%)	73 (44.5%)	8 (6.3%)	
Current-smoker	47 (16.1%)	45 (27.4%)	2 (1.6%)	
Diabetes melitus				0.070
No	185 (63.4%)	96 (58.5%)	89 (69.5%)	
Yes	107 (36.6%)	68 (41.5%)	39 (30.5%)	
Cardiovascular disease				
No	287 (98.3%)	161 (98.2%)	126 (98.4%)	1.000
Yes	5 (1.7%)	3 (1.8%)	2 (1.6%)	
Clinical stage, AJCC 8th				0.682
I	7 (2.4%)	3 (1.8%)	4 (3.1%)	
II	2 (0.7%)	2 (1.2%)	0 (0%)	
III	141 (48.3%)	80 (48.8%)	61 (47.7%)	
IV	142 (48.6%)	79 (48.2%)	63 (49.2%)	
Chemotherapy regimen				0.954
Gemcitabine plus nab-Paclitaxel	60 (20.5%)	33 (20.1%)	27 (21.1%)	
FOLFIRINOX	232 (79.5%)	131 (79.9%)	101 (78.9%)	
First line chemotherapy response				0.598
Partial response	122 (41.8%)	64 (39%)	58 (45.3%)	
Stable disease	133 (45.5%)	76 (46.3%)	57 (44.5%)	
Progressive disease	29 (9.9%)	19 (11.6%)	10 (7.8%)	
Not evaluated	8 (2.7%)	5 (3%)	3 (2.3%)	
CA 19-9, U/mL				

Initial	236.02 (1.2-140000)	163.5 (1.2-140000)	349.5 (2-123205.02)	0.010
Six months[a]	56.5 (2-291132)	40.42 (2-291132)	132 (2-140000)	0.003
Total protein, g/dl				
Initial	7 (4.3-8.7)	7.1 (4.3-8.7)	7 (5.1-8.6)	0.067
Six months[a]	7 (4.3-8.6)	7 (4.5-8.5)	6.9 (4.3-8.6)	0.153
Albumin, g/dl				
Initial	4.3 (2.9-5.1)	4.3 (2.9-5.1)	4.3 (2.9-5)	0.224
Six months[a]	4 (1.4-5.2)	4 (1.4-5.2)	3.9 (2-4.8)	0.117
Triceps skinfold thickness, mm				
Initial	10 (2-42)	8 (2-42)	12 (3-40)	<.001
Six months[a]	8 (2-30)	6.375 (2-20)	10 (2.3-30)	<.001

[a]There exist missing values in data: 26 (8.9%), 28 (9.6%), 28 (9.6%), and 55 (18.8%) of the study population for CA 19-9, total protein, albumin, and triceps skinfold thickness measured at six-month, respectively.

Abbreviations: mo, months; ECOG, Eastern Cooperative Oncology Group; AJCC, American Joint Committee on Cancer.

Table S2. Comparison of initial TSF by sex according to clinical characteristics

	Males	Females	*P* for comparison	*P* for interaction
Age, years				0.278
≤ 60	8 (6-12)	12 (10-18)	<.001	
> 60	8 (6-11.3)	11 (8-15.8)	<.001	
Body mass index, kg/m^2				0.764
Underweight < 18.5	5 (4-10.5)	8 (6-11.8)	0.217	
Normal 18.5 to < 23	8 (6-11)	12 (9-16.3)	<.001	
Overweight ≥ 23	8 (6-13)	13 (10-17)	<.001	
ECOG performance status				0.112
0	8 (6-12)	14 (10-18)	<.001	
1	7 (5-10)	10 (7.3-12.8)	0.000	
Clinical stage, AJCC 8th				0.817
I-III	8 (6-12)	12 (9-16.5)	<.001	
IV	8 (6-11)	12 (9-16.3)	<.001	
Chemotherapy regimen				0.151
Gemcitabine + nab-paclitaxel	8 (6-12)	12 (9-16.5)	0.028	
FOLFIRINOX	8 (6-11)	12 (9-16.3)	<.001	
Initial CA19-9, U/mL				0.716
≤ 34	8 (6-10)	12 (8-16)	0.001	
> 34	8 (6-12)	12 (9.3-16.8)	<.001	
Initial protein, g/dL				0.539
> 6.4	8 (6-12)	12 (9-17)	<.001	
≤ 6.4	7 (6-11)	11 (8.5-16)	<.001	
Initial albumin, g/dL				0.643
> 3.5	8 (6-12)	12 (9-17)	<.001	
≤ 3.5	6 (5.8-8.5)	10.5 (7.3-15.8)	0.026	

Data are presented as median (interquartile range).
Abbreviations: TSF, triceps skinfold thickness; ECOG, Eastern Cooperative Oncology Group; AJCC, American Joint Committee on Cancer.

Table S3. Comparison of initial albumin by sex according to clinical characteristics

	Males	Females	*P* for comparison	*P* for interaction
Age, years				0.776
≤ 60	4.4 (4-4.6)	4.3 (4-4.6)	0.564	
> 60	4.3 (3.9-4.5)	4.2 (3.9-4.4)	0.705	
Body mass index, kg/m^2				0.017
Underweight < 18.5	3.9 (3.7-4.1)	4.3 (3.9-4.6)	0.078	
Normal 18.5 to < 23	4.2 (3.9-4.5)	4.3 (4-4.5)	0.584	
Overweight ≥ 23	4.4 (4.1-4.6)	4.2 (3.9-4.4)	0.021	
ECOG performance status				0.390
0	4.3 (4.0-4.6)	4.3 (4-4.5)	0.465	
1	4.25 (3.8-4.5)	4.2 (3.9-4.4)	0.984	
Clinical stage, AJCC 8th				0.038
I-III	4.2 (3.9-4.4)	4.3 (4-4.6)	0.283	
IV	4.4 (4-4.6)	4.2 (3.9-4.4)	0.052	
Chemotherapy regimen				0.909
Gemcitabine + nab-paclitaxel	4.3 (4-4.5)	4.25 (4-4.4)	0.481	
FOLFIRINOX	4.3 (3.9-4.5)	4.3 (3.9-4.5)	0.683	
Initial CA19-9, U/mL				0.032
≤ 34	4.2 (3.9-4.5)	4.4 (4.1-4.7)	0.123	
> 34	4.3 (4-4.5)	4.2 (3.9-4.4)	0.120	
Initial protein, g/dL				0.110
> 6.4	4.4 (4.2-4.6)	4.3 (4.1-4.6)	0.176	
≤ 6.4	3.7 (3.5-4)	3.9 (3.6-4.1)	0.138	
Initial TSF, mm				0.671
> 7	4.2 (3.9-4.5)	4 (3.8-4.4)	0.269	
≤ 7	4.3 (4-4.6)	4.3 (4-4.5)	0.325	

Data are presented as median (interquartile range).
Abbreviations: ECOG, Eastern Cooperative Oncology Group; AJCC, American Joint Committee on Cancer; TSF, triceps skinfold thickness.

Table S4. Factors predicting overall survival in the study cohort

Variables	Univariate			Multivariate		
	HR	95% CI	*P*	HR	95% CI	*P*
Age, years (> 60 vs. ≤ 60)	1.08	0.84–1.40	0.55			
Sex (Female vs. Male)	1.04	0.80–1.34	0.779			
Body mass index, kg/m^2			0.875			
(Underweight vs. Normal)	0.94	0.56–1.59	0.825			
(Overweight vs. Normal)	0.93	0.72–1.21	0.612			
ECOG performance status (1 vs. 0)	1.37	1.06–1.77	0.017	1.07	0.77–1.49	0.674
Smoking			0.345			
(Ex-smoker vs. Never)	1.24	0.93–1.67	0.145			
(Current smoker vs. Never)	1.07	0.75–1.53	0.713			
Diabetes mellitus (Yes vs. No)	1.02	0.78–1.32	0.905			
Cardiovascular disease (Yes vs. No)	0.31	0.08–1.24	0.097	0.38	0.05–2.74	0.336
Clinical stage, AJCC 8th (IV vs. I-III)	1.91	1.48–2.46	<.001	1.55	1.11–2.17	0.011
Chemotherapy regimen (FOLFIRINOX vs. Gemcitabine + nab-paclitaxel)	0.61	0.45–0.83	0.001	0.66	0.42–1.04	0.071
First line chemotherapy response			<.001			<.001
(Stable disease vs. Partial response)	1.36	1.03–1.79	0.032	1.25	0.89–1.75	0.203
(Progressive disease vs. Partial response)	4.32	2.81–6.62	<.001	3.8	2.21–6.55	<.001
Initial CA 19-9, U/mL (> 34 vs. ≤ 34)	1.26	0.92–1.71	0.149			
Six-month CA 19-9, U/mL (> 34 vs. ≤ 34)	2.21	1.66–2.95	<.001	2.09	1.50–2.92	<.001
Initial albumin, g/dL (≤ 3.5 vs. > 3.5)	1.64	1.01–2.65	0.045	2.8	1.58–4.95	<.001
Albumin changes over 6 mo, g/dl (≤ -0.6 vs. > -0.6)	1.76	1.31–2.36	<.001	1.7	1.13–2.55	0.01
Initial protein, g/dL (≤ 6.4 vs. > 6.4)	1.04	0.75–1.46	0.801			
Protein changes over 6 mo, g/dL (≤ -0.9 vs. > -0.9)	2.43	1.71–3.47	<.001	2.03	1.27–3.23	0.003
Initial TSF, mm (≤ 7 vs. > 7)	1.12	0.85–1.49	0.414			
TSF changes over 6 mo, mm (≤ 0 vs. > 0)	1.56	1.13–2.16	0.007	1.51	1.08–2.12	0.017

Abbreviations: HR, hazard ratio; CI, confidence interval; ECOG, Eastern Cooperative Oncology Group; AJCC, American Joint Committee on Cancer; mo, months; TSF, triceps skinfold thickness.

Table S5. Factors predicting overall survival in males after multiple imputation

Variables	Univariate			Multivariate		
	HR	95% CI	*P*	HR	95% CI	*P*
Age, years (> 60 vs. ≤ 60)	1.07	0.75-1.51	0.716			
Body mass index, kg/m^2			0.999			
(Underweight vs. Normal)	1.01	0.40-2.52	0.985			
(Overweight vs. Normal)	1.01	0.70-1.44	0.965			
ECOG performance status (1vs. 0)	1.50	1.06-2.13	0.023	1.51	1.00-2.28	0.048
Smoking			0.100			
(Ex-smoker vs. Never)	1.58	1.04-2.42	0.033			
(Current smoker vs. Never)	1.41	0.88-2.25	0.151			
Diabetes mellitus (Yes vs. No)	0.87	0.61-1.23	0.420			
Cardiovascular disease (Yes vs. No)	0.24	0.03-1.75	0.158			
Clinical stage, AJCC 8th (IV vs. I-III)	1.97	1.39-2.79	<.001	1.89	1.22-2.93	0.005
Chemotherapy regimen						
(FOLFIRINOX vs. Gemcitabine + nab-paclitaxel)	0.66	0.44-0.99	0.044	1.09	0.64-1.87	0.741
First line chemotherapy response			<.001			0.019
(Stable disease vs. Partial response)	1.50	1.02-2.21	0.039	1.16	0.75-1.79	0.509
(Progressive disease vs. Partial response)	3.70	2.12-6.44	<.001	2.46	1.25-4.83	0.010
Initial CA 19-9, U/mL (> 34 vs. ≤ 34)	1.23	0.83-1.84	0.304			
Six-month CA 19-9, U/mL (> 34 vs. ≤ 34)	2.10	1.45-3.06	<.001	1.71	1.14-2.54	0.009
Initial albumin, g/dL (≤ 3.5 vs. > 3.5)	1.22	0.64-2.35	0.545			
Albumin changes over 6 mo, g/dL						
(≤ -0.4 vs. > -0.4)	1.41	0.94-2.13	0.096	1.25	0.81-1.90	0.307
Initial protein, g/dL (≤ 6.4 vs. > 6.4)	1.04	0.67-1.63	0.858			
Protein changes over 6 mo, g/dL						
(≤ 0.2 vs. > 0.2)	1.37	0.94-2.00	0.103			
Initial TSF, mm (≤ 7 vs. > 7)	0.94	0.66-1.34	0.740			
TSF changes over 6 mo, mm (≤ 0 vs. > 0)	1.61	1.08-2.40	0.020	1.51	0.91-2.51	0.110

Abbreviations: HR, hazard ratio; CI, confidence interval; ECOG, Eastern Cooperative Oncology Group; AJCC, American Joint Committee on Cancer; mo, months; TSF, triceps skinfold thickness.

Table S6. Factors predicting overall survival in females after multiple imputation

Variables	Univariate			Multivariate		
	HR	95% CI	*P*	HR	95% CI	*P*
Age, years (> 60 vs. ≤ 60)	1.07	0.73-1.58	0.728			
Body mass index, kg/m^2			0.797			
(Underweight vs. Normal)	0.92	0.48-1.76	0.796			
(Overweight vs. Normal)	0.86	0.56-1.34	0.509			
ECOG performance status (1 vs. 0)	1.24	0.84-1.83	0.280			
Smoking			0.679			
(Ex-smoker vs. Never)	1.09	0.47-2.51	0.839			
(Current smoker vs. Never)	0.42	0.06-3.12	0.396			
Diabetes mellitus (Yes vs. No)	1.31	0.86-1.99	0.209			
Cardiovascular disease (Yes vs. No)	0.41	0.06-3.04	0.380			
Clinical stage, AJCC 8th (IV vs. I-III)	1.82	1.23-2.68	0.003	1.52	0.91-2.52	0.109
Chemotherapy regimen						
(FOLFIRINOX vs. Gemcitabine + nab-paclitaxel)	0.56	0.35-0.89	0.014	0.59	0.33-1.06	0.075
First line chemotherapy response			<.001			<.001
(Stable disease vs. Partial response)	1.22	0.81-1.85	0.335	1.55	0.94-2.58	0.088
(Progressive disease vs. Partial response)	8.51	4.06-17.84	<.001	5.27	2.21-12.58	<.001
Initial CA 19-9, U/mL (> 34 vs. ≤ 34)	1.25	0.76-2.08	0.376			
Six-month CA 19-9, U/mL (> 34 vs. ≤ 34)	2.38	1.48-3.84	<.001	2.33	1.37-3.97	0.002
Initial albumin, g/dL (≤ 3.5 vs. > 3.5)	3.11	1.48-6.52	0.003	4.89	1.88-12.7	0.002
Albumin changes over 6 mo, g/dL						
(≤ -0.7 vs. > -0.7)	2.08	1.34-3.24	0.001	2.82	1.36-5.84	0.006
Initial protein, g/dL (≤ 6.4 vs. > 6.4)	0.87	0.52-1.45	0.591			
Protein changes over 6 mo, g/dL						
(≤ -0.9 vs. > -0.9)	2.32	1.43-3.76	0.001	1.33	0.62-2.83	0.453
Initial TSF, mm (≤ 16 vs. > 16)	0.90	0.59-1.37	0.608			
TSF changes over 6 mo, mm	1.13	0.65-1.98	0.648			

Abbreviations: HR, hazard ratio; CI, confidence interval; ECOG, Eastern Cooperative Oncology Group; AJCC, American Joint Committee on Cancer; mo, months; TSF, triceps skinfold thickness.

Table S7. Factors predicting overall survival in males in the total cohort

Variables	Univariate HR	Univariate 95% CI	Univariate *P*	Multivariate HR	Multivariate 95% CI	Multivariate *P*
Age, years (> 60 vs. ≤ 60)	1.08	0.80-1.47	0.608			
Body mass index, kg/m^2			0.315			
(Underweight vs. Normal)	1.58	0.85-2.95	0.150			
(Overweight vs. Normal)	0.97	0.70-1.33	0.834			
ECOG performance status (1vs. 0)	1.74	1.29-2.36	<.001	1.28	0.83-1.98	0.268
Smoking			0.232			
(Ex-smoker vs. Never)	1.34	0.93-1.94	0.117			
(Current smoker vs. Never)	1.34	0.90-2.01	0.147			
Diabetes mellitus (Yes vs. No)	0.81	0.59-1.10	0.180			
Cardiovascular disease (Yes vs. No)	1.37	0.64-2.93	0.420			
Clinical stage, AJCC 8th (IV vs. I-III)	2.19	1.60-2.99	<.001	1.71	1.08-2.71	0.023
Chemotherapy regimen						
(FOLFIRINOX vs. Gemcitabine + nab-paclitaxel)	0.61	0.43-0.86	0.005	0.77	0.43-1.39	0.390
First line chemotherapy response			<.001			<.001
(Stable disease vs. Partial response)	1.57	1.09-2.26	0.016	1.19	0.75-1.89	0.456
(Progressive disease vs. Partial response)	4.46	2.79-7.12	<.001	4.17	2.07-8.43	<.001
Initial CA 19-9, U/mL (> 34 vs. ≤ 34)	1.27	0.89-1.82	0.186			
Six-month CA 19-9, U/mL (> 34 vs. ≤ 34)	2.11	1.47-3.05	<.001	1.93	1.24-3.00	0.003
Initial albumin, g/dL (≤ 3.5 vs. > 3.5)	1.85	1.17-2.93	0.009	1.94	0.91-4.15	0.088
Albumin changes over 6 mo, g/dL						
(≤ -0.4 vs. > -0.4)	1.42	0.98-2.05	0.065	1.35	0.87-2.09	0.181
Initial protein, g/dL (≤ 6.4 vs. > 6.4)	1.29	0.90-1.85	0.165			
Protein changes over 6 mo, g/dL						
(≤ 0.2 vs. > 0.2)	1.36	0.94-1.98	0.101			
Initial TSF, mm (≤ 7 vs. > 7)	0.83	0.61-1.13	0.239			
TSF changes over 6 mo, mm (≤ 0 vs. > 0)	1.76	1.14-2.72	0.011	1.60	1.00-2.56	0.050

Abbreviations: HR, hazard ratio; CI, confidence interval; ECOG, Eastern Cooperative Oncology Group; AJCC, American Joint Committee on Cancer; mo, months; TSF, triceps skinfold thickness.

Table S8. Factors predicting overall survival in females in the total cohort

Variables	Univariate			Multivariate		
	HR	95% CI	*P*	HR	95% CI	*P*
Age, years (> 60 vs. ≤ 60)	1.17	0.83-1.66	0.375			
Body mass index, kg/m^2			0.654			
(Underweight vs. Normal)	1.03	0.59-1.79	0.923			
(Overweight vs. Normal)	0.84	0.56-1.25	0.382			
ECOG performance status (1 vs. 0)	1.31	0.93-1.86	0.123			
Smoking			0.559			
(Ex-smoker vs. Never)	0.83	0.37-1.89	0.659			
(Current smoker vs. Never)	0.37	0.05-2.65	0.322			
Diabetes mellitus (Yes vs. No)	1.30	0.90-1.88	0.168			
Cardiovascular disease (Yes vs. No)	0.69	0.17-2.81	0.604			
Clinical stage, AJCC 8th (IV vs. I-III)	1.95	1.37-2.77	<.001	1.49	0.94-2.37	0.088
Chemotherapy regimen						
(FOLFIRINOX vs. Gemcitabine + nab-paclitaxel)	0.61	0.41-0.92	0.017	0.64	0.36-1.12	0.119
First line chemotherapy response			<.001			0.007
(Stable disease vs. Partial response)	1.24	0.84-1.83	0.287	1.58	0.96-2.59	0.070
(Progressive disease vs. Partial response)	7.30	3.89-13.70	<.001	3.90	1.61-9.42	0.003
Initial CA 19-9, U/mL (> 34 vs. ≤ 34)	1.34	0.84-2.15	0.217			
Six-month CA 19-9, U/mL (> 34 vs. ≤ 34)	2.47	1.55-3.95	<.001	2.42	1.46-4.02	0.001
Initial albumin, g/dL (≤ 3.5 vs. > 3.5)	3.26	1.81-5.87	<.001	5.66	2.45-13.06	<.001
Albumin changes over 6 mo, g/dL						
(≤ -0.7 vs. > -0.7)	2.04	1.31-3.18	0.002	2.88	1.41-5.87	0.004
Initial protein, g/dL (≤ 6.4 vs. > 6.4)	1.20	0.79-1.82	0.397			
Protein changes over 6 mo, g/dL						
(≤ -0.9 vs. > -0.9)	2.29	1.40-3.76	0.001	1.23	0.58-2.58	0.589
Initial TSF, mm (≤ 16 vs. > 16)	0.79	0.53-1.17	0.243			
TSF changes over 6 mo, mm	1.31	0.80-2.14	0.280			

Abbreviations: HR, hazard ratio; CI, confidence interval; ECOG, Eastern Cooperative Oncology Group; AJCC, American Joint Committee on Cancer; mo, months; TSF, triceps skinfold thickness.
